# Risk Factors Associated With Mortality and Neurologic Disability After Intracerebral Hemorrhage in a Racially and Ethnically Diverse Cohort

**DOI:** 10.1001/jamanetworkopen.2022.1103

**Published:** 2022-03-15

**Authors:** Daniel Woo, Mary E. Comeau, Simone Uniken Venema, Christopher D. Anderson, Matthew Flaherty, Fernando Testai, Steven Kittner, Michael Frankel, Michael L. James, Gene Sung, Mitchell Elkind, Bradford Worrall, Chelsea Kidwell, Nicole Gonzales, Sebastian Koch, Christiana Hall, Lee Birnbaum, Douglas Mayson, Bruce Coull, Marc Malkoff, Kevin N. Sheth, Jacob L. McCauley, Jennifer Osborne, Misty Morgan, Lee Gilkerson, Tyler Behymer, Elisheva R. Coleman, Jonathan Rosand, Padmini Sekar, Charles J. Moomaw, Carl D. Langefeld

**Affiliations:** 1 Department of Neurology and Rehabilitation Medicine, University of Cincinnati College of Medicine, Cincinnati, Ohio; 2 Department of Biostatistics and Data Science, Center for Precision Medicine, Wake Forest University, Winston-Salem, North Carolina; 3 Center for Genomic Medicine, Massachusetts General Hospital, Boston; 4 Department of Neurology and Rehabilitation Medicine, University of Illinois College of Medicine, Chicago; 5 Department of Neurology, Baltimore Veterans Administration Medical Center, University of Maryland School of Medicine, Baltimore; 6 Department of Neurology, Emory University, Grady Memorial Hospital, Atlanta, Georgia; 7 Departments of Anesthesiology and Neurology, Duke University, Durham, North Carolina; 8 Neurocritical Care and Stroke Division, University of Southern California, Los Angeles; 9 Department of Neurology, Columbia University, New York, New York; 10 Department of Neurology, University of Virginia, Charlottesville; 11 Department of Neurology, University of Arizona, Tucson; 12 University of Colorado Anschutz Medical Campus, Aurora; 13 Department of Neurology, University of Miami, Miller School of Medicine, Miami, Florida; 14 Department of Neurology and Neurotherapeutics, UT–Southwestern, Dallas, Texas; 15 Department of Neurology, University of Texas at San Antonio, San Antonio; 16 Department of Neurology, Medstar Georgetown University Hospital, Washington, District of Columbia; 17 Department of Neurology and Neurosurgery, University of Tennessee Health Sciences, Memphis; 18 Department of Neurology, Yale University, New Haven, Connecticut; 19 John P. Hussman Institute for Human Genomics, University of Miami Miller School of Medicine, Miami, Florida

## Abstract

**Introduction:**

Intracerebral hemorrhage (ICH) is the most severe subtype of stroke. Its mortality rate is high, and most survivors experience significant disability.

**Objective:**

To assess primary patient risk factors associated with mortality and neurologic disability 3 months after ICH in a large, racially and ethnically balanced cohort.

**Design, Setting, and Participants:**

This cohort study included participants from the Ethnic/Racial Variations of Intracerebral Hemorrhage (ERICH) study, which prospectively recruited 1000 non-Hispanic White, 1000 non-Hispanic Black, and 1000 Hispanic patients with spontaneous ICH to study the epidemiological characteristics and genomics associated with ICH. Participants included those with uniform data collection and phenotype definitions, centralized neuroimaging review, and telephone follow-up at 3 months. Analyses were completed in November 2021.

**Exposures:**

Patient demographic and clinical characteristics as well as hospital event and imaging variables were examined, with characteristics meeting P < .20 considered candidates for a multivariate model. Elements included in the ICH score were specifically analyzed.

**Main Outcomes and Measures:**

Individual characteristics were screened for association with 3-month outcome of neurologic disability or mortality, as assessed by a modified Rankin Scale (mRS) score of 4 or greater vs 3 or less under a logistic regression model. A total of 25 characteristics were tested in the final model, which minimized the Akaike information criterion. Analyses were repeated removing individuals who had withdrawal of care.

**Results:**

A total of 2568 patients (mean [SD] age, 62.4 [14.7] years; 1069 [41.6%] women and 1499 [58.4%] men) had a 3-month outcome determination available, including death. The final logistic model had a significantly higher area under the receiver operating characteristics curve (*C* = 0.88) compared with ICH score alone (*C* = 0.76; *P* < .001). Among characteristics associated with neurologic disability and mortality were larger log ICH volume (OR, 2.74; 95% CI, 2.36-3.19; *P* < .001), older age (OR per 1-year increase, 1.04; 95% CI, 1.02-1.05; *P* < .001), pre-ICH mRS score (OR, 1.62; 95% CI, 1.41-1.87; *P* < .001), lobar location (OR, 0.22; 95% CI, 0.16-0.30; *P* < .001), and presence of infection (OR, 1.85; 95% CI, 1.42-2.41; *P* < .001).

**Conclusions and Relevance:**

The findings of this cohort study validate ICH score elements and suggest additional baseline and interim patient characteristics were associated with variation in 3-month outcome.

## Key Points

**Question:**

In addition to traditional risk factors associated with neurologic disability and mortality after intracerebral hemorrhage (ICH), are there additional risk factors, and are there differences by race or ethnicity?

**Findings:**

This cohort study including 2568 patients with ICH validated traditional risk factors associated with post-ICH mortality and neurologic disability and identified additional risk factors associated with neurologic disability and mortality, including easily measured baseline measures, such as ICH volume and location, as well as interim hospital events, such as infection.

**Meaning:**

This cohort study identified a number of novel risk factors and provided additional data supporting associations for established risk factors in estimating outcomes after ICH, which may be used both clinically and in clinical trial design.

## Teaser

This cohort study assesses risk factors associated with 3-month neurologic and mortality outcomes after intracerebral hemorrhage in a racially and ethnically diverse cohort.

## Introduction

Intracerebral hemorrhage (ICH) is the subtype of stroke with the highest disability rate among survivors. ICH has a 40% to 50% mortality rate within 30 days, 2-fold that of ischemic stroke,^1^ with only 27% of patients being functionally independent at 90 days.^2^ Black individuals have a higher overall mortality rate from ICH, likely secondary to having 2-fold the incidence rate.^3^ However, the case-fatality rate among Black individuals is lower than among White individuals, possibly owing to younger mean age among Black individuals who experience ICH.^4^ There is a paucity of information on outcomes after ICH among Hispanic patients.

The ICH score, based on 161 patients, identified age older than 80 years, lower admission Glasgow Coma Scale (GCS) score, ICH volume greater than 30 cm^3^, intraventricular hemorrhage (IVH), and infratentorial ICH as factors associated with mortality at 30 days after ICH.^5,6^ The FUNC score, based on 629 patients, identified age, GCS score, ICH location, ICH volume, and pre-ICH cognitive impairment as factors associated with disability at 90 days after ICH.^7^ Both of these scores are simple and easy to use; however, additional phenotypes have had demonstrated importance, and the populations used to develop and validate these scores had a paucity of members of racial or ethnic minority populations, such as Black and Hispanic individuals.

The importance of using a more comprehensive set of variables may affect consideration of variables in clinical trials, epidemiology, and outcomes studies. Using data from 3000 patients with ICH with equal power among White, Black, and Hispanic populations, we sought to identify additional factors associated with outcomes after ICH.

## Methods

For this multicenter cohort study, institutional review board approval was obtained at all participating centers, and informed consent was obtained from all patients or their legally authorized representative. This study is reported following the Strengthening the Reporting of Observational Studies in Epidemiology (STROBE) reporting guideline.

The methods of the Ethnic/Racial Variations of Intracerebral Hemorrhage (ERICH) study have been previously published.^8^ Briefly, the ERICH study is a multicenter study of ICH in non-Hispanic White patients, non-Hispanic Black patients, and Hispanic patients recruited at 19 centers throughout the United States. The target for enrollment was 1000 patients with ICH from each racial and ethnic group. Race and ethnicity were assessed by self-report by patient or proxy. A “hot pursuit” recruitment strategy was used rather than a retrospective patient identification method to limit survival and severity bias.^9^

Computed tomography (CT) images during hospitalization were analyzed by a centralized core. Blood samples were collected. The 2 *APOE* single nucleotide variations at amino acids 112 and 158 of the *APOE* gene were targeted individually by allele-specific probes (rs429358 and rs7412; TaqMan, ThermoFisher Scientific). All genotyping was completed at the University of Miami Miller School of Medicine. Follow-up evaluations were completed at 3, 6, and 12 months after ICH, including modified Rankin Scale (mRS),^10^ Barthel index,^11^ and quality of life measures.

### Statistical Analysis

Each variable included first passed range, category checks, and outlier detection. Each variable was tested for differences in the 3-month outcome status, with a poor outcome defined as mRS score of 4 to 6 and a good outcome defined as mRS score of 0 to 3, using a logistic regression model. The results were summarized as an odds ratio (OR) for a change of 1 unit of the variable, the corresponding 95% CI, and *P* value. In addition, the mean and SD for continuous variables and count plus percentage for categorical variables are reported. Analyses included patients with and without comfort measures only or withdrawal of care (WOC). To address the potential for prospective enrollment of patients to cause a survival bias, we performed an external validity check that compared a 6-month period of patients with ICH who were not enrolled with those who were enrolled.

The multivariable logistic regression modeling included variables with *P* < .20 and less than 5% missing data. However, because of their perceived importance, neuroimaging findings (ie, van Swieten scale,^12^ atrophy score, Graeb score^13^) and anticoagulant and antiplatelet restart data were included in the modeling despite missing data rates (approximately 10%-15%). The final models were determined using stepwise methods, with variable retention based on the lowest Akaike information criterion (AIC). The area under the receiver operating characteristic (ROC) curve (*C* statistic) was compared for the multivariate model and ICH score.^14^

Because several of the variables in the final AIC-based models did not reach a statistical threshold of *P* < .05, a second stepwise model was computed with entry and exit criteria of *P* < .05. The multivariable logistic modeling was computed including and excluding individuals who had WOC. The models’ goodness-of-fit statistics and lack of excessive influence due to being an outlier were examined. Finally, because the multivariable analyses are based on the observations with no missing data, key variables (eg, mRS) were tested for evidence of differential missingness between those observations included in the model vs excluded. Standard regression diagnostics were computed for all models reported to assure the models fit these data. This includes collinearity diagnostics (ie, variance inflation factors, condition index). The Genetic and Environmental Risk Factors for Hemorrhagic Stroke (GERFHS) study^9^ was used as a validation cohort.

To test whether the lower mortality rate among racial and ethnic groups was independent of age and ICH volume associations, we computed formal tests of race and ethnicity, age, and log volume interactions under a logistic regression model (alive or dead at 3 months as outcome). *P* values were 2-sided, and statistical significance was set at *P* < .05. Analyses were completed in November 2021.

## Results

Between July 1, 2010, and June 30, 2015, 3000 patients with spontaneous ICH were recruited across 19 centers with a preplanned distribution of 1000 White patients, 1000 Black patients, and 1000 Hispanic patients. A total of 2568 patients with ICH (mean [SD] age, 62.4 [14.7] years; 1069 [41.6%] women and 1499 [58.4%] men) who had 3-month outcome measures (85.6%), including death, form the main analyses, including 829 Black patients (32.3%), 879 Hispanic patients (34.2%), and 860 White patients (33.5%). As patients consented to genetic testing as well as follow-up, a potential survival or severity bias toward enrollment was possible. To evaluate the extent of such potential biases, each center performed a retrospective health record review on patients with spontaneous ICH who were not enrolled in their center (eTable 1 in the Supplement). This analysis found that enrolled patients were less likely to have died during hospitalization (11.4% vs 31.4%; *P* < .001), were approximately 4 years younger (mean [SD] age, 61.0 [16.5] years vs 65.0 [14.0] years; *P* < .001), and were more likely to enroll if Black (35.5% vs 32.4%) or Hispanic (32.7% vs 19.3%; *P* < .001) (eTable 1 in the Supplement). There were no significant differences in enrollment rates by sex or the major ICH risk factors, including hypertension, anticoagulant use, diabetes, hypercholesterolemia, frequent alcohol use, insurance status, and discharge of survivors to home or facility.

At 3 months, 115 patients (4.5%) had no symptoms (mRS = 0), 401 patients (15.6%) had no significant disability (mRS = 1), 414 patients (16.1%) had slight disability (mRS = 2), 383 patients (14.9%) had moderate disability (mRS = 3), 480 patients (18.7%) had moderately severe disability (mRS = 4), 210 patients (8.2%) had severe disability (mRS = 5), and 565 patients (22.0%) had died (mRS = 6). Table 1 summarizes the demographic and baseline risk factor characteristics of patients with ICH stratified by good (mRS = 0-3) vs poor (mRS = 4-6) outcomes at 3 months.

**Table 1.** Univariate Analysis of Associations of Prior History, Medications Use, Baseline Glucose, Subsequent Events, Imaging, and *APOE* E Alleles With Risk for Neurologic Disability or Mortality After ICH

Variable	Patients, No. (%)		Odds ratio (95% CI)	*P* value
	mRS 0-3 (n = 1313)	mRS 4-6 (n = 1255)		
Age, mean (SD), y	59.0 (13.3) [58.0]	66.0 (15.2) [66.0]	1.04 (1.03-1.04)	<.001
Sex				
Women	504 (38.4)	565 (45.0)	1.31 (1.12-1.54)	<.001
Men	809 (61.6)	690 (55.0)		
Race and ethnicity				
Black	453 (34.5)	376 (30.0)	1 [Reference]	NA
Hispanic	467 (35.6)	412 (32.8)	1.06 (0.88-1.29)	<.001
White	393(29.9)	467 (37.2)	1.43 (1.18-1.73)	<.001
Overall comparison	NA	NA	NA	<.001
BMI, mean (SD) [median]^a^	29.3 (6.8) [28.3]	28.5 (7.9) [27.3]	0.99 (0.97-1.00)	<.001
ICH volume, mean (SD) [median]^b^	12.2 (14.4) [6.9]	30.8 (31.5) [20.0]	1.96 (1.81-2.12)	<.001
ICH location				
Brainstem	53 (4.1)	81 (6.6)	1.57 (1.09-2.25)	.02
Cerebellum	103 (8.0)	90 (7.3)	0.90 (0.66-1.21)	.48
Deep	682 (53.1)	665 (53.9)	1 [Reference]	NA
Lobar	429 (33.4)	371 (30.1)	0.89 (0.74-1.06)	.18
Overall comparison	NA	NA	NA	.02
Primary IVH	18 (1.4)	27 (2.2)	1.54 (0.84-2.82)	.16
IVH present^c^	392 (29.9)	697 (55.5)	2.94 (2.49-3.45)	<.001
Admission GCS score, mean (SD) [median]	13.9 (2.5) [15.0]	10.8 (4.2) [12.0]	0.76 (0.74-0.79)	<.001
Prestroke mRS score, mean (SD) [median]	0.3 (0.7) [0.0]	0.9 (1.3) [0.0]	1.71 (1.57-1.87)	<.001
Medical history				
Alzheimer or dementia	31 (2.4)	149 (11.9)	5.57 (3.75-8.27)	<.001
Hypertension	1085 (82.7)	1089 (87.3)	1.44 (1.16-1.80)	.001
Anti-hypertensive drugs used, mean (SD) [median], No.	1.3 (1.5) [1]	1.5 (1.50) [1]	1.08 (1.03-1.14)	.002
Diabetes	341 (26.0)	410 (32.7)	1.38 (1.17-1.64)	<.001
High cholesterol	556 (42.7)	617 (50.3)	1.36 (1.16-1.59)	<.001
ICH	60 (4.6)	105 (8.4)	1.91 (1.37-2.64)	<.001
Ischemic stroke	126 (9.6)	198 (15.8)	1.77 (1.39-2.24)	<.001
Depression	107 (8.1)	131 (10.4)	1.31 (1.00-1.72)	.05
Malignant neoplasm	98 (7.5)	118 (9.4)	1.29 (0.97-1.70)	.08
Migraine	65 (5.0)	27 (2.2)	0.42 (0.27-0.67)	<.001
Smoking				.20
Current	232 (17.7)	209 (16.7)	0.92 (0.74-1.14)	.44
Former	411 (31.3)	382 (30.4)	0.95 (0.79-1.13)	.55
Never	668 (50.9)	655 (52.2)	1 [Reference]	NA
Unknown	2 (0.2)	9 (0.7)	4.59 (0.99-21.32)	.05
Overall comparison	NA	NA	NA	.20
Alcohol use, drinks/d				<.001
None	651 (50.2)	710 (58.5)	1 [Reference]	NA
<0-2	458 (35.3)	348 (28.7)	0.70 (0.58-0.83)	<.001
2-5	57 (4.4)	37 (3.1)	0.60 (0.39-0.91)	.02
>5	132 (10.2)	118 (9.7)	0.82 (0.63-1.07)	.15
Overall comparison	NA	NA	NA	<.001
CVD history				
Coronary CAD	167 (12.7)	198 (15.8)	1.29 (1.03-1.60)	.03
MI	59 (4.5)	77 (6.1)	1.39 (0.98-1.97)	.06
Atrial fibrillation on ECG	102 (7.8)	160 (12.7)	1.74 (1.34-2.25)	<.001
CHF	87 (6.6)	115 (9.2)	1.42 (1.06-1.90)	.02
Bypass	45 (3.4)	60 (4.8)	1.42 (0.95-2.10)	.08
Pacemaker	35 (2.7)	51 (4.1)	1.55 (1.00-2.40)	.05
AICD	10 (0.8)	17 (1.4)	1.79 (0.82-3.93)	.15
Cardiomyopathy	28 (2.1)	38 (3.0)	1.43 (0.87-2.35)	.15
Carotid endarterectomy	8 (0.6)	18 (1.4)	2.37 (1.03-5.48)	.04
PVD	29 (2.2)	39 (3.1)	1.42 (0.87-2.31)	.16
History of medication use				
Anticoagulants	128 (9.7)	178 (14.2)	1.53 (1.20-1.95)	<.001
Any antihypertensive	699 (53.2)	755 (60.2)	1.33 (1.13-1.55)	<.001
β-blocker	358 (27.3)	432 (34.4)	1.40 (1.18-1.66)	<.001
Diuretic	326 (24.8)	347 (27.6)	1.16 (0.97-1.38)	.10
α-2 adrenergic	57 (4.3)	75 (6.0)	1.40 (0.98-1.99)	.06
Statin	334 (25.4)	392 (31.2)	1.33 (1.12-1.58)	.001
SSRI	83 (6.3)	121 (9.6)	1.58 (1.18-2.11)	.002
Glucose value by life squad, mean (SD) [median]	137.3 (50.3) [127.5]	150.3 (65.9) [130.0]	1.00 (1.00-1.01)	.01

Abbreviations: AICD, automated implantable cardioverter defibrillator; BMI, body mass index; CAD, coronary artery disease; CHF, congestive heart failure; CVD, cardiovascular disease; ECG, electrocardiogram; GCS, Glasgow Coma Scale; ICH, intracerebral hemorrhage; IVH, intraventricular hemorrhage; MI, myocardial infarction; mRS, modified Rankin Scale; NA, not applicable; PVD, peripheral vascular disease; SSRI, selective serotonin reuptake inhibitor.

^a^ BMI was Winsorized to 60 for values greater than 60.

^b^ Log of ICH volume is used in *P* value and odds ratio calculation.

^c^ IVH present was not used in multivariate modeling; Graeb score (*P* = 2.63 × 10^−35^) was used instead.

A total of 76 characteristics were tested for association with good vs poor outcome using logistic regression. The ICH score (OR, 2.86; 95% CI, 2.57-3.19; *P* < .001; *C* = 0.762) and FUNC score (OR, 0.46; 95% CI, 0.43-0.50; *P* < .001; *C* = 0.757) were both associated with poor outcome and had good prognostic ability per the *C* statistic. All of the individual variables that comprise the ICH and FUNC scores were associated with poor outcome, corroborating their individual contributions to risk (Table 1). Of 76 characteristics examined, 51 were included in multivariate modeling, having met the *P* < .20 criteria (Table 1). Repeating this analysis excluding individuals who had WOC, we found 41 characteristics that met the *P* < .20 criteria (eTable 2 in the Supplement). Of 346 patients with WOC, 329 patients (95.1%) died by 3 months.

In addition to ICH and FUNC score variables, preexisting medical conditions, medication use, subsequent events, and neurological findings were also associated with poor outcome (Table 1 and Table 2). A previous history of ischemic stroke (OR, 1.77; 95% CI, 1.39-2.24; *P* < .001), atrial fibrillation (OR, 1.74; 95% CI, 1.34-2.25; *P* < .001), and β-blocker use (OR, 1.40; 95% CI, 1.18-1.66; *P* < .001) were among the medical conditions and medication use factors associated with risk of poor outcome. Although likely applied as standard of care, antihypertensive medication treatment and lowering blood pressure to a target systolic blood pressure less than 140 mm Hg was not associated with outcomes (OR, 1.25; 95% CI, 0.82-1.21; *P* = .06), and coagulopathy reversal by different methods showed mixed results (Table 3). Numerous in-hospital complications and events, such as presence of infection (OR, 3.13; 95% CI, 2.62-3.75; *P* < .001) or requiring surgical intervention (OR, 2.40; 95% CI, 1.84-3.11; *P* < .001), intracranial pressure treatment (OR, 3.97; 95% CI, 3.17-4.98; *P* < .001), intraventricular drain (OR, 3.74; 95% CI, 3.02-4.62; *P* < .001), and tracheostomy (OR, 15.82; 95% CI, 6.89-36.34; *P* < .001) were also associated with poor outcomes (Table 2). Similarly, there were numerous neuroimaging findings associated with outcomes at 3 months, including white matter hyperintensity severity (van Swieten score: OR, 1.30; 95% CI, 1.22-1.38; *P* < .001), total atrophy score (OR, 1.27; 95% CI, 1.19-1.35; *P* < .001), total intraventricular hemorrhage severity (Graeb score: OR, 1.25; 95% CI, 1.21-1.29; *P* < .001), and magnetic resonance (MR) remote diffusion weighted imaging (DWI)–positive lesions (OR, 2.46; 95% CI, 1.83-3.30; *P* < .001). We note that MR DWI-positive lesions had a high missing data rate (59% missing) and was therefore not included in the multivariable modeling.

**Table 2.** Univariate Analysis of Subsequent Events, Imaging, and Apolipoprotein E Alleles on Risk of Neurologic Disability or Mortality After ICH

Variable	Patients, No. (%)		Odds ratio (95% CI)	*P* value
	mRS 0-3 (n = 1313)	mRS 4-6 (n = 1255)		
Hematoma expansion	117 (9.3)	171 (14.3)	1.63 (1.27-2.09)	<.001
Presence of infection	238 (18.1)	514 (41.0)	3.13 (2.62-3.75)	<.001
Treatment required				
Surgical intervention	92 (7.0)	192 (15.3)	2.40 (1.84-3.11)	<.001
ICP treatment	117 (8.9)	351 (28.0)	3.97 (3.17-4.98)	<.001
Intraventricular drain	139 (10.6)	385 (30.7)	3.74 (3.02-4.62)	<.001
Tracheostomy	6 (0.5)	85 (6.8)	15.82 (6.89-36.34)	<.001
Early blood pressure lowering				
First measured SBP >140 mm Hg	1045 (79.6)	1003 (79.9)	1.00 (0.82-1.21)	.96
Antihypertensive given and lowered SBP <140 mm Hg	153 (11.7)	178 (14.2)	1.25 (0.99-1.58)	.06
Seizure as a complication	40 (3.0)	80 (6.4)	2.17 (1.47-3.19)	<.001
Clinical seizure	92 (7.0)	149 (11.9)	1.79 (1.36-2.35)	<.001
Coagulopathy reversal				
Using warfarin at stroke onset	103 (7.8)	150 (12.0)	1.27 (0.86-1.88)	.23
FFP	88 (6.7)	141 (11.2)	1.53 (1.02-2.29)	.04
Cryoprecipitate	2 (0.2)	3 (0.2)	0.80 (0.13-5.05)	.82
Platelets	142 (10.8)	197 (15.7)	1.47 (1.16-1.86)	.001
Vitamin K	84 (6.4)	116 (9.2)	0.84 (0.54-1.29)	.42
Prothrombotic complex	13 (1.0)	26 (2.1)	1.71 (0.84-3.49)	.14
Anticoagulant restart	72 (5.5)	73 (8.8)	1.67 (1.19-2.34)	.003
Dual antiplatelet restart^a^	8 (0.6)	1 (0.1)	0.20 (0.02-1.58)	.13
Neuroimaging findings				
Total van Swieten score, mean (SD)^b^	1.1 (1.3) [1.0]	1.6 (1.5) [1.0]	1.30 (1.22-1.38)	<.001
Total atrophy score, mean (SD)	1.4 (1.3) [1.0]	1.8 (1.4) [2.0]	1.27 (1.19-1.35)	<.001
Total Graeb score, mean (SD) [median]	1.2 (2.1) [0.0]	2.6 (2.9) [1.0]	1.25 (1.21-1.29)	<.001
MRI positive for microbleed^c^	155 (32.5)	101 (38.3)	1.29 (0.94-1.76)	.11
MRI DWI positive^c^	106 (16.1)	129 (32.0)	2.46 (1.83-3.30)	<.001
*APOE* genotype				
2/2	10 (0.8)	7 (0.6)	0.97 (0.34-2.75)	.95
2/3	123 (9.4)	151 (12.3)	1.70 (1.07-2.69)	.03
2/4	33 (2.5)	53 (4.3)	2.22 (1.23-4.00)	.008
3/3	786 (60.2)	696 (56.6)	1.22 (0.81-1.84)	.34
3/4	295 (22.6)	280 (22.8)	1.31 (0.85-2.01)	.22
4/4	58 (4.4)	42 (3.4)	1 [Reference]	NA
*APOE* 2 allele				
Categorical	166 (12.7)	211 (17.2)	1.42 (1.14-1.77)	.002
Continuous				
Per 1-unit increase	NA	NA	1.34 (1.09-1.65)	.005
0 copies of 2-allele	1139 (87.3)	1018 (82.8)	0.68 (0.55-0.86)	<.001
1 copy of 2-allele	156 (12.0)	204 (16.6)	1 [Reference]	NA
2 copies of 2-allele	10 (0.8)	7 (0.6)	0.54 (0.20-1.44)	.22

Abbreviations: DWI, diffusion weighted imaging; FFP, fresh frozen plasma; ICH, intracerebral hemorrhage; ICP, intracranial pressure; MRI, magnetic resonance imaging; mRS, modified Rankin Scale; NA, not applicable; SBP, systolic blood pressure.

^a^ Dual antiplatelet use is very sparse and was left out of multivariate modeling.

^b^ The van Swieten score is a measure of white matter hyperintensity severity based on computed tomography scan imaging. Graeb score is a measurement of intraventricular hemorrhage severity. MRI was evaluated when available, including microbleed and DWI burden.

^c^ MRI scores had a missing rate of more than 55% and were not used in multivariate modeling. DWI positive refers to remote DWI positive lesions separate from the hematoma.

**Table 3.** Multiple Logistic Regression of Characteristics of Patients With ICH Comparing Odds of Neurologic Disability or Mortality After ICH^a^

Variable	Estimate (SE)	Odds ratio (95% CI)	*P* value
Log of ICH volume	1.01 (0.08)	2.74 (2.36-3.19)	<.001
ICH location			
Brainstem	1.39 (0.28)	4.03 (2.34-6.95)	<.001
Cerebellum	−0.53 (0.24)	0.59 (0.37-0.94)	.03
Deep	NA	1 [Reference]	NA
Lobar	−1.53 (0.17)	0.22 (0.16-0.30)	<.001
Prestroke mRS score	0.48 (0.07)	1.62 (1.41-1.87)	<.001
Admission GCS score	−0.12 (0.02)	0.88 (0.85-0.92)	<.001
Age, per 1-y increase	0.04 (0.006)	1.04 (1.02-1.05)	<.001
Presence of infection	0.62 (0.13)	1.85 (1.42-2.41)	<.001
Hematoma expansion	0.79 (0.18)	2.20 (1.55-3.13)	<.001
Treatment required			
ICP treatment	0.74 (0.18)	2.09 (1.46-3.00)	<.001
Tracheostomy	1.84 (0.52)	6.31 (2.29-17.35)	<.001
Intraventricular drain	0.64 (0.19)	1.89 (1.30-2.74)	<.001
Total Graeb score	0.11 (0.03)	1.12 (1.05-1.18)	<.001
Female sex	0.43 (0.13)	1.53 (1.19-1.98)	.001
Prior use of α-2 adrenergic	0.81 (0.27)	2.24 (1.32-3.82)	.003
Total van Swieten score	0.14 (0.05)	1.15 (1.04-1.26)	.005
Medical history			
Alzheimer or dementia	0.92 (0.30)	2.51 (1.40-4.51)	.002
Migraine	−0.97 (0.38)	0.38 (0.18-0.80)	.01
Ischemic stroke	0.48 (0.20)	1.62 (1.11-2.37)	.01
Diabetes	0.34 (0.14)	1.40 (1.07-1.83)	.01
Cardiomyopathy	0.77 (0.35)	2.15 (1.08-4.27)	.03
ICH	0.46 (0.27)	1.59 (0.94-2.68)	.08
Hypertension	0.22 (0.18)	1.24 (0.88-1.75)	.22
Total atrophy score	0.15 (0.06)	1.16 (1.03-1.31)	.02
*APOE* 2 allele	0.36 (0.17)	1.43 (1.02-2.01)	.04
Clinical seizure	0.45 (0.23)	1.56 (1.00-2.43)	.05
Prior use of anticoagulants	−0.24 (0.20)	0.79 (0.53-1.16)	.23

Abbreviations: GCS, Glasgow Coma Scale; ICH, intracerebral hemorrhage; ICP, intracranial pressure; mRS, modified Rankin Scale; NA, not applicable.

^a^ Model based on Akaike information criteria. Odds ratios were computed for a change of 1 for continuous variables. Analysis includes patients who had withdrawal of care.

The multiple logistic regression model that minimized the AIC score retained 25 of the patient characteristics and had a significantly higher area under the ROC curve (*C* = 0.88) compared with either the ICH score alone (*C* = 0.76) or the FUNC score alone (*C* = 0.76), with *P* < .001 for the difference in *C* statistics between the logistic regression model and ICH score and FUNC score. Among the characteristics associated with poor outcome were ICH volume (OR, 2.74; 95% CI, 2.36-3.19; *P* < .001), location of ICH (eg, lobar: OR, 0.22; 95% CI, 0.16-0.30; *P* < .001), pre-ICH mRS score (OR, 1.62; 95% CI, 1.41-1.87; *P* < .001), admission GCS score (OR, 0.88; 95% CI, 0.85-0.92; *P* < .001), age (OR per 1-year increase, 1.04; 95% CI, 1.02-1.05; *P* < .001), and presence of infection (OR, 1.85; 95% CI, 1.42-2.41; *P* < .001). Comparing the model that included patients with WOC (Table 3) with the model that excluded patients with WOC (eTable 2 in the Supplement) found a similar list of characteristics. A set of parallel models based on statistical significance are provided in eTable 3 and eTable 4 in the Supplement and found comparable results.

In addition, we performed an evaluation of the overall model in an external cohort (GERFHS) (Table 4). The power of each variable and concordance are provided in eAppendix 1 and eAppendix 2 in the Supplement. There was a high concordance rate for direction of association and β coefficients; however, with less power, not every finding was validated. Yet the application of the variable set identified a high ROC value (GERFHS: *C* = 0.935; ERICH: *C* = 0.882). Complementary to this, we performed an analysis of risk factors for death by 3 months and identified ICH volume, atrophy, prestroke disability, older age, brainstem location, IVH severity, history of infection, and history of diabetes to be associated with mortality (eTable 5 in the Supplement).

**Table 4.** Multiple Logistic Regression Model of Characteristics of Patients With ICH Comparing Odds of Neurologic Disability or Mortality After ICH in the Genetic and Environmental Risk Factors for Hemorrhagic Stroke Study External Data Set for Variables Identified in the Final Multiple Logistic Regression Model^a^

Variable	Estimate (SE)	Odds ratio (95% CI)^b^	*P* value
Log of ICH volume	0.86 (0.13)	2.36 (1.83-3.03)	<.001
ICH location			
Brainstem	0.65 (0.48)	1.92 (0.75-4.90)	.17
Cerebellum	−0.06 (0.41)	0.94 (0.42-2.10)	.88
Lobar	−1.51 (0.31)	0.22 (0.12-0.40)	<.001
Prestroke mRS score	0.53 (0.11)	1.69 (1.37-2.10)	<.001
Admission GCS score	−0.41 (0.05)	0.66 (0.60-0.73)	<.001
Age, per 1-y increase	0.04 (0.01)	1.04 (1.02-1.07)	<.001
Presence of infection	0.55 (0.26)	1.73 (1.05-2.86)	.03
Treatment required			
ICP treatment	1.31 (0.56)	3.71 (1.24-11.11)	.02
Tracheostomy^c^	13.39 (863.2)	NA	.99
Intraventricular drain	1.39 (0.55)	4.02 (1.36-11.87)	.01
IVH score (used in place of total Graeb score)	0.04 (0.03)	1.05 (0.99-1.10)	.11
Female sex	−0.03 (0.24)	0.97 (0.61-1.54)	.90
Prior use of α-2 adrenergic	0.43 (0.44)	1.54 (0.65-3.66)	.33
Total van Swieten score	0.23 (0.13)	1.26 (0.97-1.63)	.09
Medical history			
Alzheimer or dementia	0.07 (0.39)	1.07 (0.50-2.28)	.85
Migraine	−0.99 (0.82)	0.37 (0.07-1.85)	.23
Ischemic stroke	−0.45 (0.34)	0.64 (0.33-1.24)	.18
Diabetes	0.23 (0.26)	1.26 (0.76-2.10)	.37
Cardiomyopathy	1.14 (0.62)	3.11 (0.92-10.52)	.07
ICH	0.44 (0.52)	1.56 (0.56-4.30)	.39
Hypertension	−0.28 (0.29)	0.76 (0.43-1.34)	.34
Total atrophy score	0.07 (0.14)	1.07 (0.81-1.41)	.63
Clinical seizure	0.27 (0.47)	1.31 (0.53-3.28)	.56
Prior use of anticoagulants	0.38 (0.27)	1.46 (0.85-2.49)	.17

Abbreviations: GCS, Glasgow Coma Scale; GERFHS, Genetic and Environmental Risk Factors for Hemorrhagic Stroke; ICH, intracerebral hemorrhage; ICP, intracranial pressure; IVH, intraventricular hemorrhage; mRS, modified Rankin Scale; NA, not applicable; ROC, receiver operating characteristic.

^a^ GERFHS model area under the ROC curve *C* = 0.935; The corresponding *C* statistic for the original ERICH model was *C* = 0.882. Data on hematoma expansion were not available from the GERFHS study.

^b^ Odds ratios were computed for a change of 1 unit for continuous variables. Analyses includes patients who had withdrawal of care.

^c^ Given the rarity of tracheostomy and the magnitude of the estimate and SE, the corresponding odds ratio and 95% CI are unestimable.

Finally, we tested whether the mortality rates across races and ethnicities were independent of the associations of age and ICH volume. Specifically, we tested for an age × race and ethnicity interaction and a log volume × race and ethnicity interaction under a logistic model, modeling death. There was no evidence that the association of age differed by ethnicity. However, the association of log volume with outcome did differ by race and ethnicity (log-volume for Black patients: OR, 2.43; 95% CI, 2.01-2.93; *P* < .001; log-volume for Hispanic patients: OR, 2.23; 95% CI, 1.86-2.69; *P* < .001; log-volume for White patients: OR, 1.74; 95% CI, 1.51-2.01; *P* < .001). Thus, although log volume was associated with death for all races and ethnicities, the association was greatest in Black and Hispanic patients.

We performed an external validity check that compared a 6-month period of patients with ICH who were not enrolled into the study with those who were enrolled. Mortality was higher in those not enrolled; however, no major differences were identified in sex, history of hypertension, diabetes, anticoagulant use, frequent alcohol use, or discharge to home, rehabilitation, nursing facility, or hospice.

## Discussion

This cohort study in a racially and ethnic balanced population identified additional variables to be considered in study design and clinical practice, in addition to validating variables included in the ICH and FUNC scores. Whereas infratentorial location of ICH is a factor included in the ICH score, we found a marked difference between cerebellar and brainstem ICH in their associations with 3-month outcomes. Additional baseline markers of white matter hyperintensity, atrophy, and degree of intraventricular hemorrhage can also be readily determined by baseline CT examination. Prestroke disability had a significant association with outcomes and while we confirmed the importance of prior cognitive impairment in the FUNC score, a number of other medical history variables had similar associations.

In addition to these baseline variables, subsequent hospital events, most of which would not be available at baseline, had significant associations with outcomes and may be important targets for intervention. Strikingly, the development of any infection (eg, urinary tract, pneumonia) had a similar association with outcomes as hematoma expansion or requiring treatment for increased intracranial pressure.

One interesting finding of our study was that race and ethnicity variables dropped out of the final model. Several studies have reported lower mortality rates for Black patients and Hispanic patients vs White patients.^15,16^ These lower mortality rates may be secondary to residual confounding from younger age at stroke.

Several studies have reported that elevated glucose levels may be associated with worse outcome after ICH,^17,18^ but glucose levels may also reflect worse severity. In our evaluation, after controlling for other factors, including the volume of ICH, elevated glucose at admission was no longer statistically significantly associated with 3-month outcomes. However, a prior history of diabetes remained within the model and should likely be included in future analyses.

WOC behavior is a critical aspect of outcomes studies. Some studies have found that Black and Hispanic populations have a racial- or ethnicity-specific protective association against mortality or worse outcomes. For example, a study by Ormseth et al^19^ suggested that Hispanic patients are less likely to have WOC than Black and White patients with similar ICH scores. This may affect mortality rates artificially compared with a true biologic effect. However, we found that after controlling for all other variables, including WOC, there was no specific association of race or ethnicity with disability risk.

When analyses that include patients with WOC were compared with analyses that excluded patients with WOC, the overall Graeb intraventricular hemorrhage score was replaced by presence of IVH, but the remaining variables were retained and in the same direction of association. This suggests that WOC may confound mortality rates, but the behavior is appropriate to the other factors of ICH severity.

The FUNC score previously identified that lobar ICH was associated with better outcomes than deep ICH.^20^ Our findings were highly concurrent with this prior report. The ICH score listed infratentorial location as associated with worse outcomes but did not differentiate supratentorial into deep and lobar ICH. Both ICH and FUNC score did not differentiate brainstem from cerebellum, and we found that cerebellar ICH was associated with less severe outcomes than brainstem ICH.

It is critical to note that we described procedures as *required*, such as *intraventricular drain required*. As patients were more ill, the treatment was likely provided appropriately rather than the procedure itself causing worse outcomes. Similarly, patients who required treatment for increased intracranial pressure likely reflect greater severity of stroke rather than treatment leading to worse outcomes. However, it is important to note that placement of intraventricular catheters may also increase the risk of infection, which we found was associated with worse outcomes.

In addition to the factors previously described, we found several simple measures on the initial CT, including white matter lesion burden, Graeb score, and atrophy, to be associated with disability at 3 months. Further evaluation is needed to determine which levels of each may contribute to redefining a future score. White matter hyperintensity severity is easily measured on baseline scans; previous work has suggested that white matter hyperintensity is associated with microbleeds and outcomes.^21,22^ Although presence of IVH (vs absence) has been well established as a risk factor, we found that increasing severity of IVH (according to the IVH score) was associated with increased risk of poor outcomes. Thus, outcome scores, such as the ICH and FUNC scores, may be further refined by use of IVH scores.^23^

A notable absence as a risk factor for poor outcomes was anticoagulant use at time of admission or INR, both of which were examined.^24,25^ Once controlling for the larger hemorrhage volume and higher rates of hematoma expansion, prior use of anticoagulants dropped out of the final model, which suggests that the greater risk of poor outcome is captured by those variables alone. Multiple different classes of medications were evaluated, but ultimately, the pattern of associations suggests that hypertension itself, rather than a particular class, may be the overall factor associated with outcomes.

Blood pressure–lowering and anticoagulant reversal medications did not meet criteria for the final models but were likely applied as standard of care. As this study is not a randomized clinical trial, these results should not influence practice.

The major advantages of the ICH and FUNC scores are their simplicity and ease of use. Improvements may be made by combining their elements along with other baseline events, as well as interim events. The purpose of this study is to present the relative value of additional variables. Such information may be clinically useful to treating physicians to improve on estimation of outcomes beyond the traditional scores. One study by Hwang et al found^26^ that physician estimations of outcomes were higher than either the ICH or FUNC score.

### Limitations

This study has some limitations. One limitation is that patients were prospectively enrolled, and there may be a survival bias. However, in population-based or case studies, a functional evaluation at follow-up would be difficult to perform with a large sample size without consent. Additionally, race and ethnicity were defined by self-report of the patient or proxy when the patient was unable to respond. Views on race and ethnicity have rapidly changed, and while the method of self-report is standard, it does not capture the complex nature of race and ethnicity from a perspective of exposures to structural racism, biases, and complex family structure. Future studies should consider definitions that better capture these complex issues.

## Conclusions

The findings of this cohort study support the associations of the ICH and FUNC scores with neurologic disability or mortality after ICH. However, we identified additional important factors associated with outcomes from historical, baseline imaging, and subsequent events that could increase our understanding of the recovery process and risk of poor outcomes. The ERICH study is exploring whether these results will enable the development of a more comprehensive ICH recovery score that accounts for different races and ethnicities with increased prognostic ability.

## Notes

Supplement. **eTable 1.** External Validity Check Comparing Health Record Abstraction Data From Enrolled Patients to 6 Months of Nonenrolled Patients With ICH **eTable 2.** Multiple Logistic Regression of Characteristics of Patients With ICH Comparing Good vs Poor Outcome After ICH Excluding Those With Withdrawal of Care **eTable 3.** Multiple Logistic Regression of Characteristics of Patients With ICH Comparing Good vs Poor Outcome After ICH Including Those With Withdrawal of Care **eTable 4.** Multiple Logistic Regression of Characteristics of Patients With ICH Comparing Good vs Poor Outcome After ICH Based on Statistical Significance Instead of AIC Criteria **eAppendix 1.** Power Analysis for Genetic and Environmental Risk Factors for Hemorrhagic Stroke (GERFHS) Study **eAppendix 2.** Concordance Scatterplot of β Coefficients From ERICH and GERFHS Logistic Regression Models for Modified Rankin Scores of 3 or Less vs Greater Than 3 for Variables in the Final ERICH Model **eTable 5.** Multiple Logistic Regression Model of Characteristics of Patients With ICH Comparing for Death in ERICH Study
