# Supplementary material for: Risk Factors Associated With Mortality and Neurologic Disability After Intracerebral Hemorrhage in a Racially and Ethnically Diverse Cohort
Source: JAMA Netw Open. 2022 Mar 15;5(3):e221103. doi: 10.1001/jamanetworkopen.2022.1103 (PMC8924717; doi:10.1001/jamanetworkopen.2022.1103)
Supplement: Supplement. — eTable 1. External Validity Check Comparing Health Record Abstraction Data From Enrolled Patients to 6 Months of Nonenrolled Patients With ICH eTable 2. Multiple Logistic Regression of Characteristics of Patients With ICH Comparing Good vs Poor Outcome After ICH Excluding Those With Withdrawal of Care eTable 3. Multiple Logistic Regression of Characteristics of Patients With ICH Comparing Good vs Poor Outcome After ICH Including Those With Withdrawal of Care eTable 4. Multiple Logistic Regression of Characteristics of Patients With ICH Comparing Good vs Poor Outcome After ICH Based on Statistical Significance Instead of AIC Criteria eAppendix 1. Power Analysis for Genetic and Environmental Risk Factors for Hemorrhagic Stroke (GERFHS) Study eAppendix 2. Concordance Scatterplot of β Coefficients From ERICH and GERFHS Logistic Regression Models for Modified Rankin Scores of 3 or Less vs Greater Than 3 for Variables in the Final ERICH Model eTable 5. Multiple Logistic Regression Model of Characteristics of Patients With ICH Comparing for Death in ERICH Study [file jamanetwopen-e221103-s001.pdf]

Supplemental Online Content

Woo D, Comeau ME, Venema SU, et al. Risk factors associated with mortality and neurologic disability after intracerebral hemorrhage in a racially and ethnically diverse cohort. *JAMA Netw Open*. 2022;5(3):e221103. doi:10.1001/jamanetworkopen.2022.1103

eTable 1. External Validity Check Comparing Health Record Abstraction Data From Enrolled Patients to 6 Months of Nonenrolled Patients With ICH

eTable 2. Multiple Logistic Regression of Characteristics of Patients With ICH Comparing Good vs Poor Outcome After ICH Excluding Those With Withdrawal of Care

eTable 3. Multiple Logistic Regression of Characteristics of Patients With ICH Comparing Good vs Poor Outcome After ICH Including Those With Withdrawal of Care

eTable 4. Multiple Logistic Regression of Characteristics of Patients With ICH Comparing Good vs Poor Outcome After ICH Based on Statistical Significance Instead of AIC Criteria

eAppendix 1. Power Analysis for Genetic and Environmental Risk Factors for Hemorrhagic Stroke (GERFHS) Study

eAppendix 2. Concordance Scatterplot of β Coefficients From ERICH and GERFHS Logistic Regression Models for Modified Rankin Scores of 3 or Less vs Greater Than 3 for Variables in the Final ERICH Model

eTable 5. Multiple Logistic Regression Model of Characteristics of Patients With ICH Comparing for Death in ERICH Study

This supplemental material has been provided by the authors to give readers additional information about their work.

© 2022 Woo D et al. *JAMA Network Open.*

eTable 1. External Validity Check Comparing Health Record Abstraction Data From Enrolled Patients to 6 Months of Nonenrolled Patients With ICH

	**Enrolled (N=2873)**	**Not Enrolled (N=388)**	**P-value**
Age, mean (SD)	61.0 (16.5)	65 (14.4)	P<0.001
Female	1140 (42.3%)	156 (40.3%)	0.84
Race/Ethnicity			
• Black	955 (35.5%)	121 (32.4%)	
• Hispanic	881 (32.7%)	72 (19.3%)	
• White	855 (31.8%)	180 (48.3%)	P<0.001
PMH			
• Hypertension	2005 (82.3%)	297 (80.0%)	0.29
• Diabetes	700 (28.8%)	94 (25.7%)	0.21
• Hypercholesterolemia	763 (33.3%)	107 (29.6%)	0.16
• Anticoagulant Use	339 (12.2%)	51 (14.4%)	0.24
• Heavy Drinker	267 (11.6%)	38 (10.6%)	0.46
Discharge to:			
• Home/Relative	736 (31.5%)	79 (30.7%)	
• Rehabilitation	870 (37.3%)	96 (37.4%)	
• SNF/Assisted Living	381 (16.3%)	41 (16.0%)	
• Hospice	89 (3.8%)	18 (7.0%)	
• Other	258 (11.1%)	23 (9.0%)	
• Died	327 (11.4%)	56 (31.4%)	0.12

© 2022 Woo D et al. *JAMA Network Open.*

eTable 2. Multiple Logistic Regression of Characteristics of Patients With ICH Comparing Good vs Poor Outcome After ICH Excluding Those With Withdrawal of Care

Variable	Estimate (Standard Error)	P-value	Odds Ratio (95% CI)
Log of ICH Volume	0.98 (0.08)	P<0.001	2.66 (2.27-3.12)
ICH Location		P<0.001	
Brainstem (ref=Deep)	1.07 (0.30)	P<0.001	2.92 (1.62-5.27)
Cerebellum (ref=Deep)	-0.53 (0.25)	0.03	0.59 (0.36-0.96)
Lobar (ref=Deep)	-1.58 (0.18)	P<0.001	0.21 (0.15-0.29)
Pre-stroke mRS	0.46 (0.07)	P<0.001	1.59 (1.37-1.84)
Presence of Infection	0.65 (0.14)	P<0.001	1.91 (1.45-2.50)
Age (years)	0.03 (0.006)	P<0.001	1.03 (1.02-1.04)
Admission GCS	-0.10 (0.02)	P<0.001	0.9 (0.86-0.94)
Tracheostomy Required	2.00 (0.52)	P<0.001	7.42 (2.70-20.36)
Female	0.50 (0.14)	P<0.001	1.64 (1.26-2.14)
Hematoma Expansion	0.69 (0.19)	P<0.001	2.00 (1.38-2.89)
ICP Treatment Required	0.67 (0.19)	P<0.001	1.95 (1.34-2.83)
Prior Use of Alpha2 Adrenergic	0.88 (0.27)	0.001	2.41 (1.41-4.11)
Total Graeb score	0.09 (0.03)	0.003	1.10 (1.03-1.16)
Total van Swieten score	0.14 (0.05)	0.006	1.15 (1.04-1.27)
Intraventricular Drain Required	0.55 (0.20)	0.006	1.73 (1.17-2.56)
History of Diabetes	0.38 (0.14)	0.007	1.47 (1.11-1.94)
Total Atrophy score	0.16 (0.06)	0.01	1.17 (1.03-1.33)
History of Alzheimer's or Dementia	0.76 (0.32)	0.02	2.14 (1.15-3.99)
History of Migraine	-0.98 (0.41)	0.03	0.38 (0.17-0.84)
History of Ischemic Stroke	0.42 (0.20)	0.04	1.52 (1.03-2.25)
APOE 2 Allele	0.32 (0.18)	0.08	1.38 (0.97-1.97)
History of ICH	0.48 (0.27)	0.08	1.61 (0.95-2.74)
Clinical seizure	0.40 (0.24)	0.09	1.50 (0.94-2.39)
History of Cardiomyopathy	0.61 (0.37)	0.09	1.85 (0.90-3.77)
Prior Use of Anticoagulants	-0.34 (0.22)	0.11	0.71 (0.47-1.08)
History of Hypertension	0.22 (0.19)	0.23	1.25 (0.87-1.80)

© 2022 Woo D et al. *JAMA Network Open.*

[A] Model based on AIC criteria. Odds ratios (OR) computed for a change of one for continuous variables or relative to a reference group for discrete variables. Analysis *excludes* patients that had withdrawal of care.

© 2022 Woo D et al. *JAMA Network Open.*

eTable 3. Multiple Logistic Regression of Characteristics of Patients With ICH Comparing Good vs Poor Outcome After ICH Including Those With Withdrawal of Care

Variable	Estimate (Standard Error)	P-value	Odds Ratio (95% CI)
Log of ICH Volume	1.00 (0.08)	P<0.001	2.71 (2.34-3.14)
ICH Location		P<0.001	
Brainstem (ref=Deep)	1.37 (0.27)	P<0.001	3.95 (2.31-6.74)
Cerebellum (ref=Deep)	-0.50 (0.23)	0.03	0.6 (0.39-0.94)
Lobar (ref=Deep)	-1.48 (0.16)	P<0.001	0.23 (0.17-0.31)
Age (years)	0.04 (0.005)	P<0.001	1.04 (1.03-1.05)
Pre-stroke mRS	0.54 (0.07)	P<0.001	1.71 (1.49-1.96)
Admission GCS	-0.12 (0.02)	P<0.001	0.88 (0.85-0.92)
Presence of Infection	0.63 (0.13)	P<0.001	1.88 (1.46-2.43)
Hematoma Expansion	0.84 (0.18)	P<0.001	2.31 (1.63-3.27)
ICP Treatment Required	0.745 (0.18)	P<0.001	2.11 (1.49-3.00)
Total van Swieten score	0.18 (0.05)	P<0.001	1.19 (1.09-1.31)
Total Graeb score	0.10 (0.03)	P<0.001	1.11 (1.05-1.17)
Tracheostomy Required	1.87 (0.51)	P<0.001	6.46 (2.37-17.59)
Intraventricular Drain Required	0.58 (0.18)	0.002	1.78 (1.24-2.55)
History of Diabetes	0.40 (0.13)	0.003	1.49 (1.15-1.93)
Prior Use of Alpha2 Adrenergic	0.81 (0.27)	0.003	2.25 (1.32-3.82)
History of Ischemic Stroke	0.56 (0.19)	0.003	1.76 (1.21-2.56)
History of Alzheimer's or Dementia	0.82 (0.29)	0.005	2.28 (1.29-4.04)
Female	0.33 (0.13)	0.009	1.39 (1.09-1.78)
History of Migraine	-0.99 (0.38)	0.01	0.37 (0.18-0.79)

[A] Model based on statistical significance (stepwise modeling with P<0.05 to enter or remain in the model). Odds ratios (OR) computed for a change of one for continuous variables or relative to a reference group for discrete variables. Analysis includes patients that had withdrawal of care.

© 2022 Woo D et al. *JAMA Network Open.*

eTable 4. Multiple Logistic Regression of Characteristics of Patients With ICH Comparing Good vs Poor Outcome After ICH Based on Statistical Significance Instead of AIC Criteria

Variable	Estimate (Standard Error)	P-value	Odds Ratio (95% CI)
Log of ICH Volume	0.98 (0.08)	P<0.001	2.66 (2.28-3.12)
ICH Location		P<0.001	
Brainstem (ref=Deep)	1.10 (0.29)	P<0.001	2.99 (1.68-5.33)
Cerebellum (ref=Deep)	-0.57 (0.24)	0.02	0.57 (0.36-0.90)
Lobar (ref=Deep)	-1.59 (0.17)	P<0.001	0.20 (0.15-0.29)
Age (years)	0.04 (0.005)	P<0.001	1.04 (1.03-1.05)
Pre-stroke mRS	0.49 (0.07)	P<0.001	1.63 (1.42-1.89)
Presence of Infection	0.67 (0.14)	P<0.001	1.96 (1.50-2.56)
Admission GCS	-0.10 (0.02)	P<0.001	0.90 (0.87-0.94)
Tracheostomy Required	2.01 (0.51)	P<0.001	7.49 (2.76-20.36)
Total van Swieten score	0.18 (0.05)	P<0.001	1.20 (1.09-1.31)
Hematoma Expansion	0.69 (0.19)	P<0.001	2.00 (1.39-2.89)
ICP Treatment Required	0.68 (0.19)	P<0.001	1.98 (1.38-2.86)
History of Diabetes	0.44 (0.14)	0.001	1.56 (1.19-2.04)
Prior Use of Alpha2 Adrenergic	0.88 (0.27)	0.001	2.42 (1.41-4.13)
Female	0.41 (0.13)	0.001	1.51 (1.17-1.95)
Total Graeb score	0.08 (0.03)	0.006	1.08 (1.02-1.15)
Intraventricular Drain Required	0.50 (0.19)	0.01	1.65 (1.13-2.42)
History of Migraine	-0.97 (0.40)	0.02	0.38 (0.17-0.84)
History of Ischemic Stroke	0.45 (0.20)	0.02	1.57 (1.07-2.32)
History of ICH	0.58 (0.27)	0.03	1.79 (1.06-3.02)
History of Alzheimer's or Dementia	0.68 (0.31)	0.03	1.97 (1.07-3.61)

[A] Model based on statistical significance (stepwise modeling with P<0.05 to enter or remain in the model). Odds ratios (OR) computed for a change of one for continuous variables or relative to a reference group for discrete variables. Analysis *excludes* patients that had withdrawal of care.

© 2022 Woo D et al. *JAMA Network Open.*

eAppendix 1. Power Analysis for Genetic and Environmental Risk Factors for Hemorrhagic Stroke (GERFHS) Study

Power analysis for the logistic regression model with the outcome modified Rankin Scores ≤3 vs. >3 was computed for each variable in the final ERICH model that was present in the *Genetic and Environmental Risk Factors for Hemorrhagic Stroke* (GERFHS) study. The GERFHS study is composed of 1007 self-reported non-Hispanic white ICH cases, but no other races/ethnicities. Power was computed for the observed effect size (odds ratio) in the ERICH model and again assuming a regression to the mean to 75% of the ERICH effect size. The expected number of variables that should replicate was computed (i.e., the sum of the power estimates) for the original and regressed to the mean estimates (see footnote).

Variable	Observed Odds Ratio	Power	75% of Odds Ratio	Power at 75% Odds Ratio
**Log of ICH Volume**	2.74	1.00	2.13	1.00
**Location - Brainstem**	4.03	0.90	2.84	0.74
**Location - Cerebellum**	0.59	0.56	0.67	0.34
**Location - Lobar**	0.22	1.00	0.32	1.00
**Pre-stroke mRS**	1.62	1.00	1.44	1.00
**Admission GCS**	0.88	1.00	0.91	0.99
**Age (years)**	1.04	1.00	1.03	1.00
**Presence of Infection**	1.85	0.96	1.59	0.80
**Hematoma Expansion**	2.20	0.88	1.81	0.68
**ICP Treatment Required**	2.09	0.95	1.74	0.78
**IVH Score (used in place of Total Graeb score)**	1.12	0.97	1.09	0.83
**Tracheostomy Required**	6.31	0.90	3.98	0.76
**Intraventricular Drain Required**	1.89	0.90	1.61	0.70
**Female**	1.53	0.82	1.38	0.58
**History of Alzheimer's or Dementia**	2.51	0.79	2.00	0.58
**Use of Alpha2 adenergic**	2.24	0.56	1.83	0.37
**Total van Swieten score**	1.15	0.75	1.11	0.51
**History of Migraine**	0.38	0.79	0.48	0.53
**History of Ischemic Stroke**	1.62	0.54	1.44	0.35
**History of Diabetes**	1.40	0.54	1.29	0.34
**Total Atrophy score**	1.16	0.80	1.12	0.56
**History of Cardiomyopathy**	2.15	0.31	1.78	0.21
**Presence of APOE-2 allele**	1.43	0.39	1.31	0.25
**Clinical Seizure**	1.56	0.38	1.40	0.24
**History of ICH**	1.59	0.30	1.41	0.20
**History of Hypertension**	1.24	0.19	1.18	0.13

© 2022 Woo D et al. *JAMA Network Open.*

**Prior Use of Anticoagulants**	0.79	0.20	0.83	0.13

Expected number of variables to replicate with the full ERICH effect size is 19.3.
Expected number of variables to replicate with regression of the mean effect size of 0.75 is 13.4.

© 2022 Woo D et al. *JAMA Network Open.*

eAppendix 2. Concordance Scatterplot of β Coefficients From ERICH and GERFHS Logistic Regression Models for Modified Rankin Scores of 3 or Less vs Greater Than 3 for Variables in the Final ERICH Model

The concordance scatterplot of the respective beta coefficients β coefficients from ERICH and GERFHS logistic regression models for modified Rankin Scores ≤3 vs. >3 generally shows that the coefficients are in the same direction and relatively comparable across the two datasets. The exceptions (i.e., discordant directions) are the variables with the lowest power (anti-coagulant, power=0.20; history of hypertension, power=0.19; and history of ischemic stroke, power=0.54).

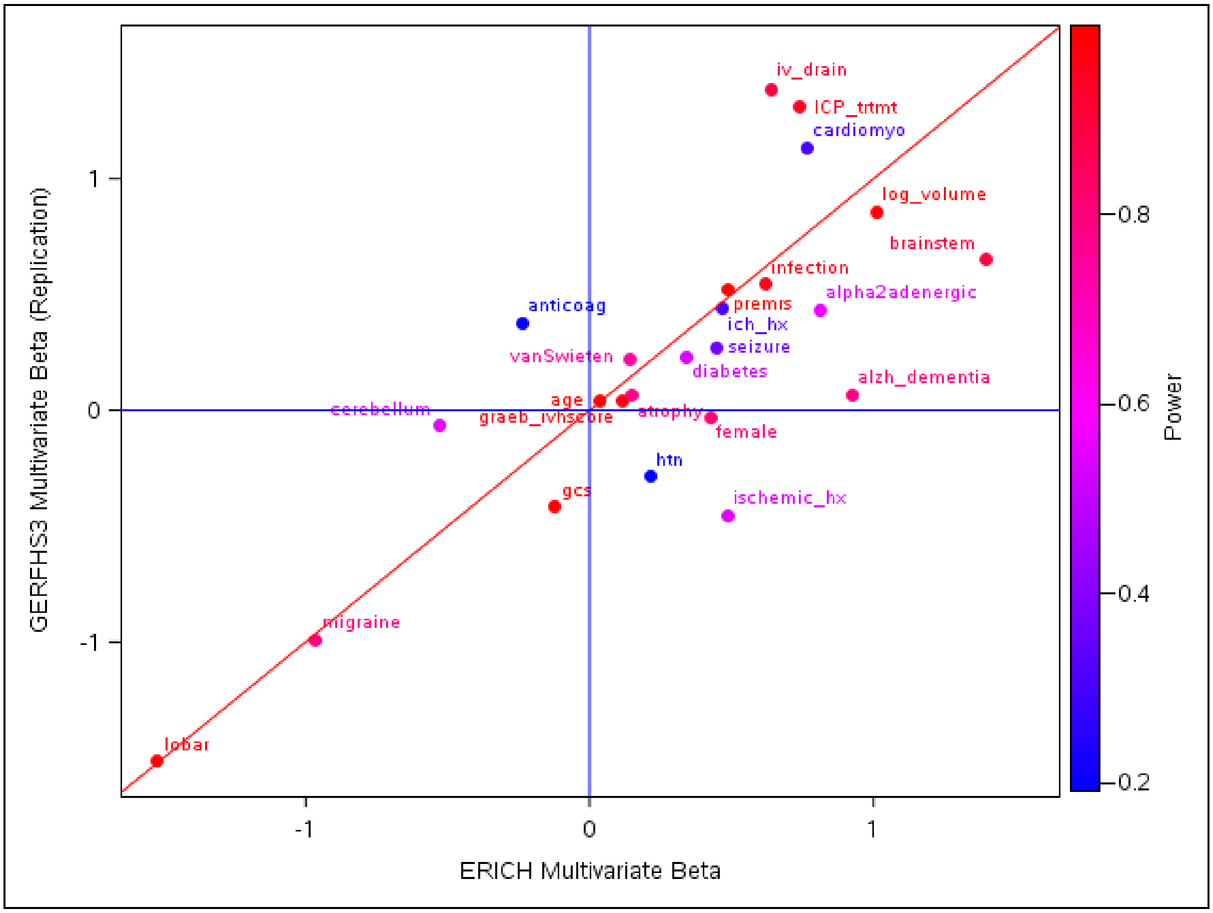

© 2022 Woo D et al. *JAMA Network Open.*

eTable 5. Multiple Logistic Regression Model of Characteristics of Patients With ICH Comparing for Death in ERICH Study

Variable	Estimate (Standard Error)	P-value	Odds Ratio (95% CI)
**Log of ICH Volume**	0.62 (0.14)	P<0.001	1.87 (1.43-2.43)
**Total Atrophy score**	0.43 (0.11)	P<0.001	1.54 (1.24-1.91)
**Pre-stroke mRS**	0.32 (0.09)	P<0.001	1.38 (1.16-1.63)
**Age (years)**	0.04 (0.01)	P<0.001	1.04 (1.01-1.06)
**Total Graeb score**	0.12 (0.05)	0.009	1.13 (1.03-1.24)
**Presence of Infection**	0.59 (0.23)	0.01	1.80 (1.15-2.82)
**History of Diabetes**	0.60 (0.24)	0.01	1.82 (1.15-2.89)
**ICH Location**		0.01	
**Brainstem (ref=Deep)**	1.35 (0.46)	0.004	3.84 (1.55-9.52)
**Cerebellum (ref=Deep)**	0.31 (0.38)	0.42	1.36 (0.64-2.89)
**Lobar (ref=Deep)**	-0.39 (0.29)	0.18	0.68 (0.39-1.19)
**Race/ethnicity**		0.01	
**White (ref=Black)**	-0.40 (0.27)	0.14	0.67 (0.39-1.14)
**Hispanic (ref=Black)**	-0.86 (0.29)	0.003	0.42 (0.24-0.75)
**ICP Treatment Required**	0.60 (0.31)	0.06	1.81 (0.98-3.36)
**Anticoagulant restart (including 3-mo F/U)**	-0.70 (0.39)	0.07	0.50 (0.23-1.06)
**Prior use of Beta Blocker**	0.38 (0.23)	0.09	1.47 (0.94-2.30)
**History of High Cholesterol**	-0.38 (0.24)	0.12	0.69 (0.43-1.10)
**History of Malignancy**	0.43 (0.32)	0.19	1.53 (0.81-2.88)
**History of MI**	-0.64 (0.49)	0.19	0.53 (0.20-1.38)

[A] Model based on AIC criteria. Odds ratios (OR) for death computed for a change of one for continuous variables or relative to a reference group for discrete variables. Analysis includes patients that had withdrawal of care.

© 2022 Woo D et al. *JAMA Network Open.*
